# Supplementary material for: Polyketide synthase gene clusters in the ericoid mycorrhizal fungus Oidiodendron maius: genome-wide prediction, expression profiling, and functional characterization of knockout mutants
Source: Front Plant Sci. 2026 Sep 16;17:1924430. doi: 10.3389/fpls.2026.1924430 (PMC13623903; doi:10.3389/fpls.2026.1924430)
Supplement: Supplementary file 1 [file SupplementaryFile1.zip › Supplementary Materials/Supplementary Folder 1.zip!/Oidma1_AssemblyScaffolds_Repeatmasked/knownclusterblast/cluster48/jgi.p_Oidma1_136635_mibig_hits.html]

| MIBiG Protein | Description | MIBiG Cluster | MiBiG Product | % ID | % Coverage | BLAST Score | E-value |
| --- | --- | --- | --- | --- | --- | --- | --- |
| EAW09121.1 | zinc-binding\_dehydrogenase\_family\_oxidoreductase,\_putative | BGC0000983 | Cytochalasin\_biosynthetic\_gene\_cluster | 40 | 98.1233243968 | 263 | 4.5e-70 |
| AGO86659.1 | equisetin\_enoylreductase | BGC0001255 | Equisetin\_biosynthetic\_gene\_cluster | 40 | 97.0509383378 | 252 | 6.1e-67 |
| XP\_001220461.1 | hypothetical\_protein | BGC0001182 | Chaetoglobosins\_biosynthetic\_gene\_cluster | 38 | 100.0 | 250 | 4e-66 |
| AAD34554.1 | enoyl\_reductase | BGC0000089 | Lovastatin\_biosynthetic\_gene\_cluster | 38 | 100.268096515 | 231 | 1.9e-60 |
| ABA02243.1 | dehydrogenase | BGC0000098 | Monacolin\_K\_biosynthetic\_gene\_cluster | 38 | 100.0 | 230 | 3.2e-60 |
| BAC20562.1 | enoyl\_reductase | BGC0000039 | Compactin\_biosynthetic\_gene\_cluster | 37 | 98.3914209115 | 225 | 1e-58 |
| CCT72378.1 | related\_to\_C.carbonum\_toxD\_protein | BGC0001305 | Fujikurins\_biosynthetic\_gene\_cluster | 35 | 96.7828418231 | 218 | 1.7e-56 |
| ADN43684.1 | DmbC | BGC0001136 | Desmethylbassianin\_biosynthetic\_gene\_cluster | 36 | 103.485254692 | 216 | 6.3e-56 |
| AKC54419.1 | trans\_enoyl-reductase | BGC0001218 | Fumosorinone\_biosynthetic\_gene\_cluster | 36 | 101.876675603 | 216 | 6.3e-56 |
| CAL69596.1 | hypothetical\_protein | BGC0001049 | Tenellin\_biosynthetic\_gene\_cluster | 36 | 103.485254692 | 210 | 3.5e-54 |
| CBF80481.1 | Enoyl\_reductase\_(Eurofung) | BGC0000959 | Aspyridone\_biosynthetic\_gene\_cluster | 35 | 98.927613941 | 195 | 1.2e-49 |
| BAQ25464.1 | zinc-binding\_dehydrogenase\_family\_protein | BGC0001280 | Betaenone\_C\_/\_betaenone\_A\_biosynthetic\_gene\_cluster | 37 | 102.144772118 | 188 | 1.4e-47 |
| CBF73513.1 | conserved\_hypothetical\_protein | BGC0001121 | Orsellinic\_acid\_biosynthetic\_gene\_cluster | 25 | 103.217158177 | 70 | 4.3e-12 |
| EAA59568.1 | hypothetical\_protein | BGC0000057 | F9775\_biosynthetic\_gene\_cluster | 25 | 103.217158177 | 70 | 4.3e-12 |
